# Effect of postoperative radiotherapy in women with localized pure mucinous breast cancer after lumpectomy: a population-based study

**DOI:** 10.1186/s13014-022-02082-7

**Published:** 2022-07-07

**Authors:** Qiuping Mo, Yongzhen Wang, JinLan Shan, Xiaochen Wang

**Affiliations:** 1 General Surgery, Cancer Center, Department of Breast Surgery, Zhejiang Provincial People’s Hospital (Affiliated People’s Hospital, Hangzhou Medical College), Hangzhou, Zhejiang China; 2 Department of Breast Surgery, Ninghai Maternal and Child Health Hospital, Ningbo, Zhejiang China; 3 Department of Breast Surgery, Women’s Hospital, Zhejiang University School of Medicine, Hangzhou, Zhejiang China; 4 Department of Breast Surgery, Second Affiliated Hospital, Zhejiang University School of Medicine, Hangzhou, Zhejiang China

**Keywords:** Breast neoplasm, Radiotherapy, Adjuvant, Lumpectomy, Prognosis, Mucinous

## Abstract

**Purpose:**

Pure mucinous breast cancer is a rare subtype of invasive breast cancer with favorable prognosis, in which the effect of postoperative radiotherapy remains unclear. We aimed to investigate the prognostic value of postoperative radiotherapy in women with localized pure mucinous breast cancer after lumpectomy.

**Methods:**

We conducted a retrospective cohort study to compare the effectiveness of postoperative radiotherapy (RT) and omitting postoperative radiotherapy (non-RT) in patients with first primary T1-2N0M0 (T ≤ 3 cm) pure mucinous breast cancer who underwent lumpectomy between 1998 and 2015 using the Surveillance, Epidemiology, and End Results (SEER) database. Breast cancer-specific survival (BCSS) was compared between RT and non-RT groups using Kaplan–Meier method and Cox proportional hazards regression model. Propensity score matching (PSM) was carried out to balance cohort baselines. In addition, an exploratory analysis was performed to verify the effectiveness of RT in subgroup patients.

**Results:**

Of 7832 eligible patients, 5352 (68.3%) underwent lumpectomy with postoperative RT, 2480 (31.7%) received lumpectomy without postoperative RT. The median follow-up duration was 92 months. The median age was 66 years in the RT group and 76 years in the non-RT group.The 15-year BCSS was 94.39% (95% CI, 93.08% to 95.35%) in the RT group versus 91.45%(95% CI, 88.93% to 93.42%) in the non-RT group (*P* < 0.001). The adjusted hazard ratio for BCSS was 0.64 (95% CI, 0.49 to 0.83; *P* = 0.001) for RT group versus non-RT group. After propensity score matching, similar results were yielded. Adjuvant RT reduced the 15-year risk of breast cancer death from 7.92% to 6.15% (*P* = 0.039). The adjusted hazard ratio for BCSS were 0.66 (95%CI, 0.47 to 0.92; *P* = 0.014) for RT group versus non-RT group. The benefit of RT was well consistent across subgroup patients.

**Conclusion:**

Among women with T1-2N0M0 (tumor size ≤ 3 cm) pure mucinous breast cancer, the addition of RT after lumpectomy was significantly associated with a reduced incidence of breast cancer death compared with non-RT, and the magnitude of benefit may be modest. This suggests that postoperative RT is recommended in the treatment of localized pure mucinous breast cancer.

## Introduction

Mucinous breast carcinoma accounting for approximately 1 to 6% of all breast cancer is divided into two pathological subtypes: pure mucinous breast cancer and mix mucinous breast carcinoma [1]. Pure mucinous breast cancer exclusively consists of tumor tissue with extracellular mucin production over 90%, whereas mix mucinous breast cancer usually mixes infiltrating ductal epithelial component with mucinous areas covering from 50 to 90% [2]**.** The comparisons of biological features and clinical prognosis have been identified previously among pure mucinous breast cancer, mix mucinous breast carcinoma and invasive breast cancer of no special type [1, 3–13]. Pure mucinous breast cancer usually occurs in elderly patients, especially in postmenopausal women [8]. The tumor size of pure mucinous breast cancer ranges from less than 1 cm to more than 20 cm, with an average of 3 cm [14]. On account of fewer genetic mutations, pure mucinous breast cancer has a stabilized luminal A phenotype with higher expression of hormone receptor and a lower rate of positive human epidermal growth factors 2 (HER-2) [5, 15, 16]. A mechanical barrier made of abundant pools of extracellular mucus around cellular island restricts carcinoma cell invasion, leading to less axillary lymph node or distant metastases. Axillary node involvement, although rare, appears to be the worst prognostic factor followed by tumor size, age, progesterone receptor(PR), HER-2 status and nuclear grade [3, 17–20]. It has reported that the 5-year, 10-year disease-free survival (DFS) were up to 94% [1], 92% [9] for patients with node-negative pure mucinous breast cancer, respectively. Hence, pure mucinous breast cancer presents distinct clinicopathological characteristics with especially favorable prognosis.

At present, the recommendations of locoregional treatment for patients with operable pure mucinous breast cancer from the latest National Comprehensive Cancer Network are the same as that for patients with typical breast cancer [21]. However, it is difficult to evaluate the effect of local regional treatment on survival outcome in prospective cohort studies or randomized trials owing to the relatively low incidence rate and a limited follow-up prognosis of pure mucinous breast cancer. Guidelines on radiotherapy of pure mucinous breast cancer are extrapolated from evidence based on other common invasive breast cancer. Although scholars have done some retrospective studies, the effect of postoperative radiotherapy in patiens with pure mucinous breast cancer is uncertain so far. Previous study showed that adjuvant radiotherapy was an independent protective factor for both overall survival (OS) and BCSS in patients with pure mucinous breast cancer. However, this retrospective study was hetetogeneous in nature because inclusion criteria involved in advanced patients, and the cohort included mastectomy and lumpectomy [22]. A recent SEER research presented that postoperative radiotherapy following lumpectomy improved the 10-year BCSS rates from 94.5 to 97.6% in patients aged ≥ 65 years diagnosed with T1–2N0 and hormone receptor-positive pure mucinous breast cancer. Yet regrettably, patients aged < 65 years were not included in this study. Besides, patients with tumor size larger than 3 cm were more likely to receive endocrine therapy, which may confuse results [23]. Obviously, it is necessary to adequately assess individualized roles of postoperative RT in this special subtype of breast carcinoma. Hence, we proceeded to a large population-based study using SEER to investigate the effect of postoperative RT on BCSS in women undergoing lumpectomy with T1-2N0M0 (tumor size ≤ 3 cm) stage pure mucinous breast cancer.

## Methods

### Patients population

This retrospective study was performed utilizing SEER database (November 2018 submission) which released cancer data from 18 registries of national cancer institute and covered approximately 28% of the US population [24]. A case-listing session was derived from SEER*Stat version 8.3.5.

**Fig. 1:**
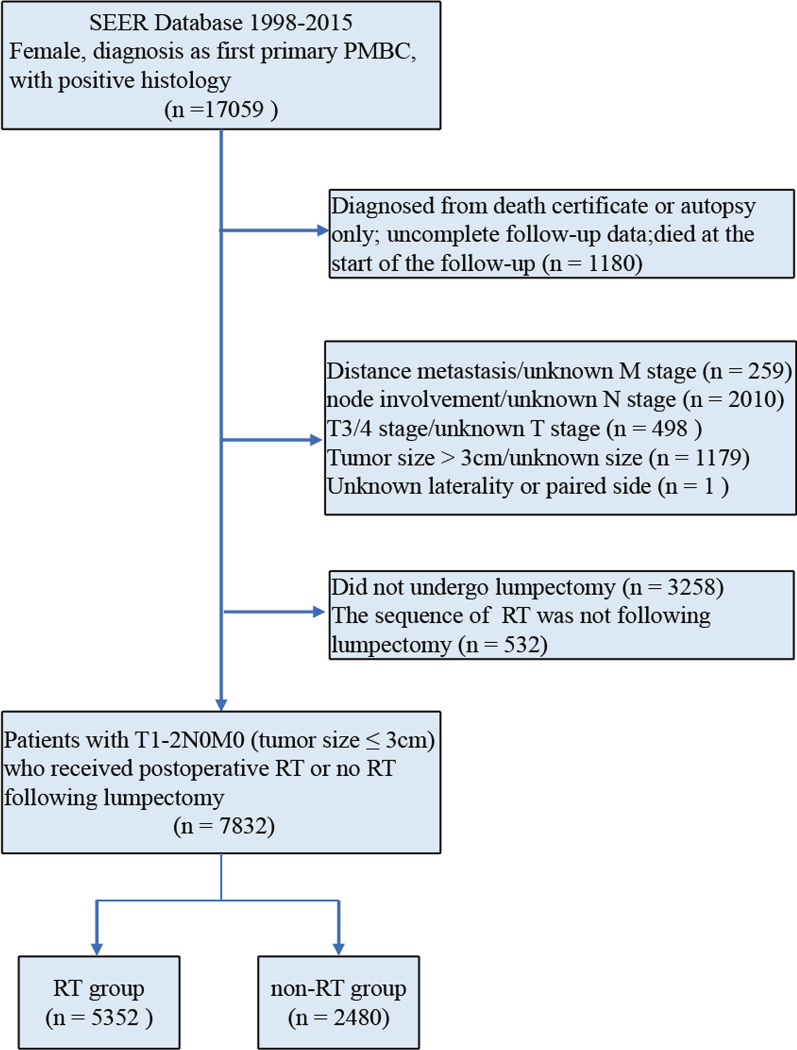
Patient selection diagram

We selected all female cases of histological diagnosed first primary pure mucinous breast cancer with the International Classification of Diseases for Oncology, 3rd Revision (ICD-O-3) code 8480/3 from January 1998 through December 2015. Patients with T1-2N0M0 (tumor size less than 3 cm) stage were eligible. And patients were required to receive lumpectomy with or without postoperative RT. The exclusion criteria were listed as follows: diagnosed from death certificate or autopsy only; no active or complete follow-up data; died at the start of the follow-up; unknown T, N, M stage; with nodal positive disease or metastases disease at diagnosis; without operation or unknown surgery; without RT or unknown RT; non-postoperative radiotherapy; bilateral cancer or unknown laterality; unknown tumor size. The flowchart for patient selection was shown in Fig. 1.

### Study covariates

According to administrations of lumpectomy and postoperative RT, a total of 7832 eligible patients under the inclusion criteria were stratified into RT group and non-RT group. We subsequently reviewed variable information of each case on patient baseline demographics, such as age at diagnoses, year of diagnoses, race, marital status at diagnoses. Then, tumor clinicopathological characteristics, including tumor laterality, tumor grade, T stage, tumor size, estrogen receptor (ER) and PR status, were extracted. Among them, the T stage was adjusted by the 6th American Joint Committee on Cancer (AJCC) TNM Staging System. Tumor grade was categorized into four levels on the biasis of the degree of differentiation: grade I, well differentiated; grade II, moderately differentiated; grade III, poorly differentiated; grade IV, undifferentiated or anaplastic. Borderline ER/PR status defined as having 1–10% positivity by immunohistochemistry were merged into positive ER/PR status [25, 26]. In this study, we did not evaluate HER-2 status because of lacking data before 2010.

### Statistical analysis

Categoric variables were compared across treatment groups using the Pearson chi-squared test, and continuous variables were analyzed by two independent sample t-tests or Wilcoxon rank sum test. The primary endpoint of this study was BCSS. BCSS was defined as an interval from the data of pure mucinous breast cancer diagnosis to death as a result of breast cancer. Using Kaplan–Meier survival analysis, BCSS were estimated with log-rank tests in unmatched groups and matched groups. Hazard Ratio (HR) and 95% confidence interval (95%CI) were calculated by Cox proportional hazards model to estimate the effect of RT. The multivariable Cox proportional hazards regression analysis incorporated variables that were significant or approximately significant in univariate analyses. The proportional-hazards assumption was checked based on Schoenfeld residuals after fitting a Cox model. And all of the Cox models obeyed the proportional risk hypothesis. PSM was used to control confounding bias in the retrospective study. Propensity scores of being receipt of RT were calculated by using a multivariable logistic regression model. The independent variables are being those that were statistically significant for correlation with treatment modality. Patients treated with RT were matched 1:1 to patients managed without RT on propensity scores by using nearest neighbor matching algorithm. The threshold value of Caliper matching was set to 0.2. A standardized difference of less than 0.1 was considered an indifferent imbalance between comparison groups. Further, exploratory analysis and tests of interaction were undertaken to evaluate the effect of adjuvant RT among subgroups according to patient and tumor characteristics.

Statistical analyses were performed with SPSS, version 24.0 (SPSS Inc., Chicago, IL, USA) and STATA, version 15 (Stata Corp., College Station, TX, USA). Two-tailed *P* < 0.05 was considered statistically significant.

## Results

### Patient demographics and tumor characteristics

**Fig. 2:**
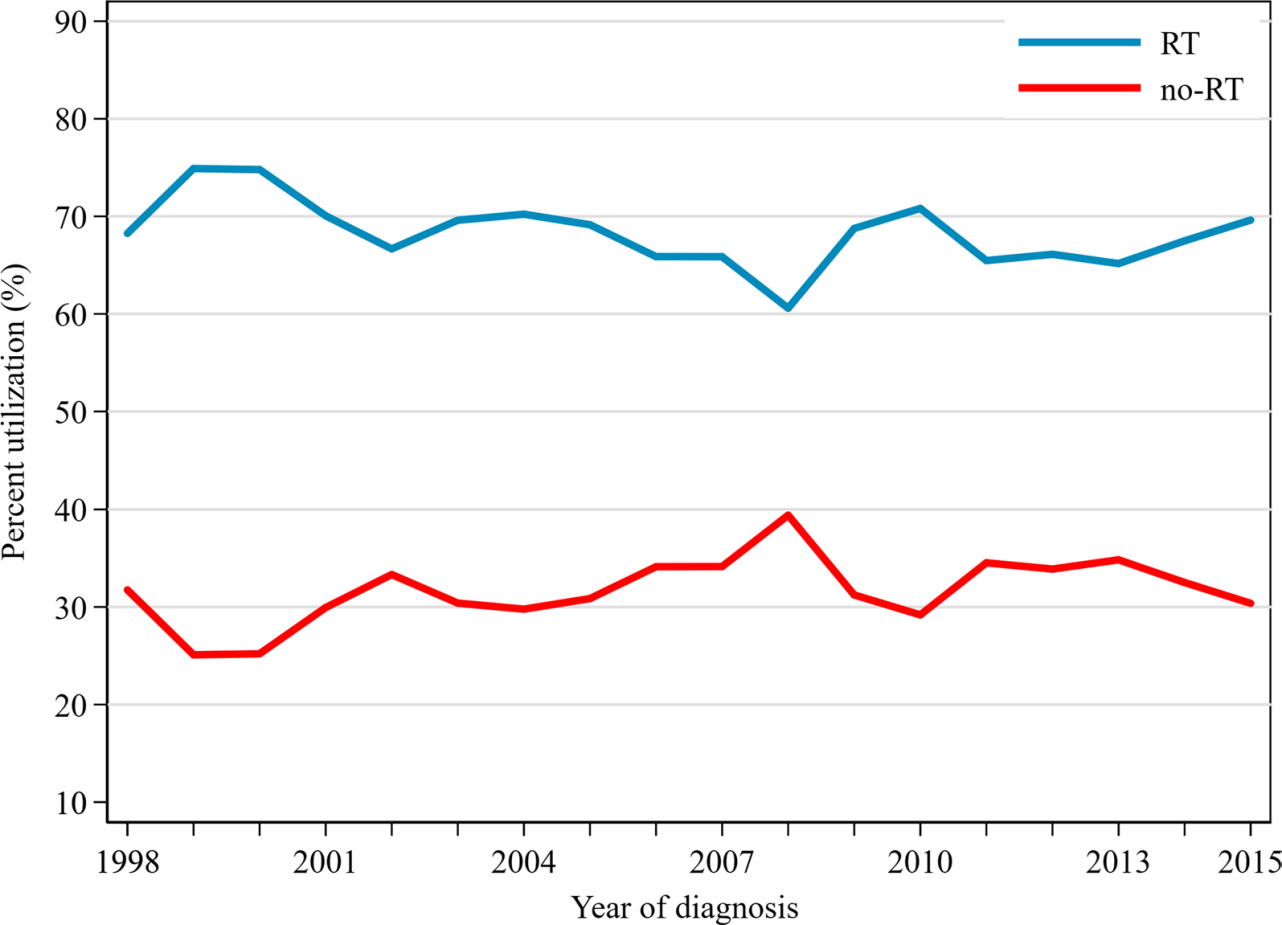
Utilization of postoperative radiotherapy versus omission over time in patients with T1-2N0M0(tumor size ≤ 3 cm) pure mucinous breast cancer from SEER Database, 1998–2015

Comparisons of patient demographics and tumor characteristics between RT and non-RT group were summarized in Table 1. A total of 7832 eligible patients with pure mucinous breast cancer were identified in the cohort (mean [SD] age, 67.1 [13.3] years), of whom 5352 (68.3%) received lumpectomy and postoperative RT, 2480(31.7%) were treated with lumpectomy without RT. Among patients underwent lumpectomy, those who received RT were on average 9 years younger than those who did not (*P* < 0.001). The median age (interquartile range) was 66 years (56–74) in RT group and 76 years (65–83) in non-RT group. The main pathological feature of patients was hormone receptor positive (91.9%) and well differentiated (55.5%). The minority of patients received chemotherapy(7.8%). There was no significant difference between treatment groups in tumor size (*P* = 0.433). There was little change in the utilization of postoperative RT through the period between 1998 and 2015(Fig. 2). In order to eliminate the imbalance between groups that may affect results, PSM was subsequently conducted. After PSM between the RT group and non-RT group, 2149 pairs were generated. The distribution of covariates was well balanced between propensity-matched groups (Table 2).

**Table 1:** Demographic and tumor characteristics among all patients with pure mucinous breast cancer

	RT		Non-RT		P*
Characteristics	No	%	No	%	
Patients	5352	68.3	2480	31.7	
*Age of diagnosis, years*					
Mean (SD)	64.4 (12.5)		72.8 (13.3)		< 0.001
Median (IQR)	66.0 (56.0–74.0)		76.0 (65.0–83.0)		< 0.001
< 50	749	14.1	194	7.8	< 0.001
50–59	1008	18.8	232	9.4	
60–69	1495	27.9	386	15.6	
≥ 70	2100	39.2	1668	67.2	
*Era of diagnosis*					
1998–2004	2027	37.9	845	34.1	0.002
2005–2009	1515	28.3	772	31.1	
2010–2015	1810	33.8	863	34.8	
Race					
White	4292	80.2	2079	83.8	< .001
Black	453	8.5	225	9.1	
Other^a^	607	11.3	176	7.1	
*Marital status*					
Married	2892	54.0	984	39.7	< 0.001
Non-married^b^	680	12.7	258	10.4	
DSW^c^	1680	30	1106	44.6	
Unknown	172	3.2	132	5.3	
Laterality					
Left	2782	52.0	1317	53.1	0.354
Right	2570	48.0	1163	46.9	
*Tumor size (T stage), cm*					
Mean (SD)	1.4 (0.7)		1.4 (0.7)		0.092
Median (IQR)	1.3 (0.9–1.8)		1.3 (0.9–1.8)		0.139
≤ 1.0 (T1)	2034	38.0	909	36.7	0.488
1.1–2.0 (T1)	2456	45.9	1170	47.2	
2.1–3.0 (T2)	862	16.1	401	16.1	
*Tumor grade*					
I	2977	55.6	1366	55.1	0.002
II	1478	27.6	627	25.3	
III	135	2.5	52	2.1	
IV	8	0.2	4	0.2	
unknown	754	14.1	431	17.4	
*ER status*					
Positive	5002	93.5	2176	87.7	< 0.001
Negative	88	1.6	30	1.2	
Unknown	262	4.9	274	11.0	
*PR status*					
Positive	4503	84.1	1953	78.8	< 0.001
Negative	510	9.5	206	8.3	
Unknown	339	6.4	321	12.9	
*Chemotherapy*					
No/Unknown	4854	90.7	2371	95.6	< 0.001
Yes	498	9.3	109	4.4	

*RT* radiotherapy, *SD* standard deviation, *IQR* interquartile range; DSW, divorced, separated and widowed

*Categoric variables were analyzed by the Pearson x^2^ test, and continuous variables (age, tumor size) were analyzed by the t tests or Mann–Whitney tests

^a^Including Asian or Pacific Islander, American Indian, Alaska Native and unknown race

^b^Including unmarried or domestic partner, single (never married)

^c^Including divorced, separated and widowed

**Table 2:** Demographic and tumor characteristics among propensity-matched population with pure mucinous breast cancer

	RT		Non-RT		Standardized difference
Characteristics	No	%	No	%	
Patients	2149	50.0	2149	50.0	
*Age of diagnosis, years*					
Mean (SD)	70 (11.9)		71 (12.9)		0.081
< 50	163	7.6	194	9.0	0.050
50–59	233	10.8	232	10.8	0.000
60–69	463	21.5	380	17.7	0.096
≥ 70	1290	60.0	1343	62.5	0.051
*Era of diagnosis*					
1998–2004	792	36.9	731	34.0	0.061
2005–2009	585	27.2	673	31.3	0.090
2010–2015	772	35.9	745	35.3	0.011
*Race*					
White	1769	82.3	1782	82.9	0.015
Black	186	8.7	202	9.4	0.026
Other	194	9.0	165	7.7	0.047
*Marital status*					
Married	932	43.3	956	44.5	0.022
Non-married	215	10.0	227	10.6	0.020
DSW	902	42.0	880	40.9	0.022
Unknown	100	4.7	86	4.0	0.033
*Laterality*					
Left	1124	52.3	1139	53.0	0.014
Right	1025	47.7	1010	47.0	0.014
*Tumor size (T stage), cm*					
Mean (SD)	1.4 (0.7)		1.4 (0.7)		0.000
≤ 1.0 (T1)	817	38.0	822	38.3	0.006
1.1–2.0 (T1)	985	45.8	1009	47.0	0.024
2.1–3.0 (T2)	347	16.1	318	14.8	0.036
*Tumor grade*					
I	1164	54.2	1212	56.4	0.044
II	566	26.3	558	26.0	0.007
III	45	2.1	46	2.1	0.000
IV	4	0.2	4	0.2	0.000
unknown	370	17.2	329	15.3	0.052
*ER status*					
Positive	1912	89.0	1952	90.8	0.060
Negative	34	1.6	30	1.4	0.012
Unknown	203	9.4	167	7.8	0.058
*PR status*					
Positive	1732	80.6	1754	81.6	0.027
Negative	182	8.5	186	8.7	0.005
Unknown	235	10.9	209	9.7	0.039
*Chemotherapy*					
No/Unknown	2019	94.0	2040	94.9	0.039
Yes	130	6.0	109	5.1	0.039

*RT* radiotherapy, *SD* standard deviation, *DSW* divorced, separated and widowed

### Survival analyses of BCSS

**Fig. 3:**
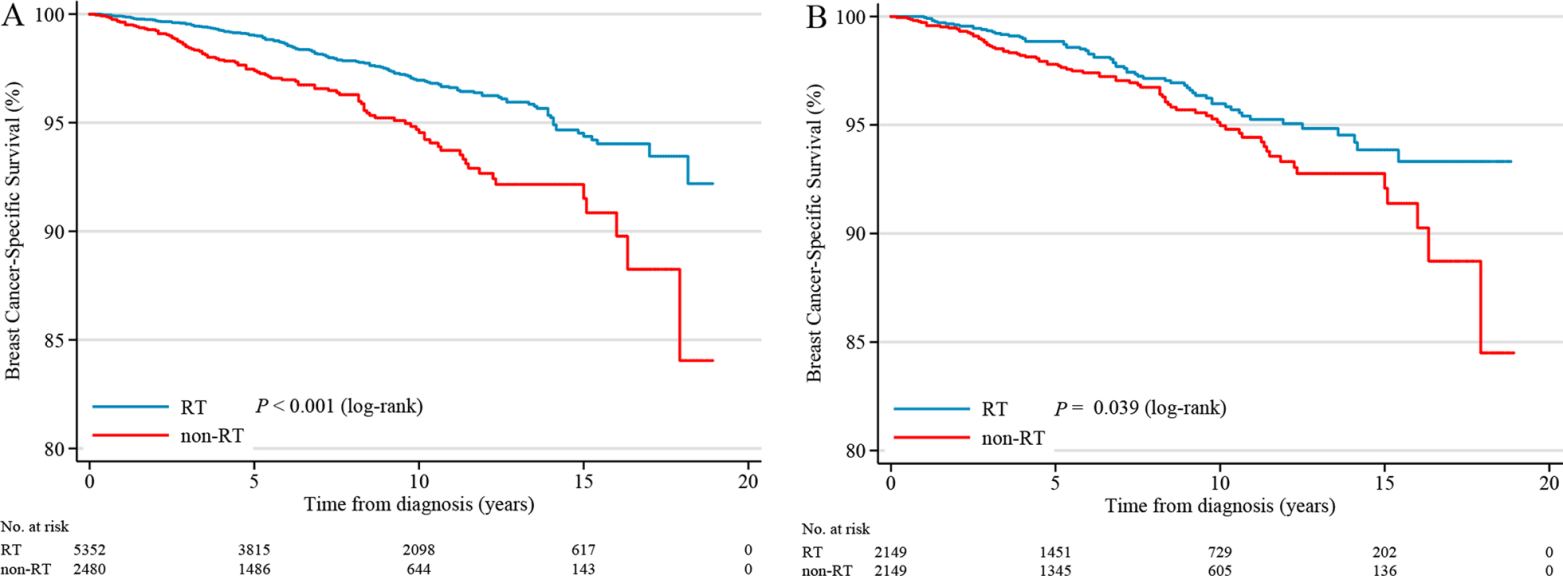
Kaplan–Meier curves comparing BCSS between treatment groups for (**A**) all patients; (**B**) propensity-matched patients

Overall, the median follow-up time was 92 months (interquartile range, 48 to142 months), and 239 breast cancer-special deaths were observed. The Kaplan–Meier survival estimate showed that 5-year, 10-year, 15-year BCSS rates were 99.01% (95% CI, 98.68% to 99.26%), 96.95% (95% CI, 96.29% to 97.49%), 94.39% (95% CI, 93.08% to 95.35%) for patients treated with RT respectively, whereas the corresponding were 97.38% (95% CI, 96.57–98.01%), 94.50% (95% CI, 93.08–95.64%), 91.45%(95% CI, 88.93–93.42%) for non-RT respectively. The difference between RT and non-RT curve was statistically significant (log-rank test, *P* < 0.001; Fig. 3A). The univariate Cox proportional hazards regression model showed the HR of BCSS for RT versus non-RT was 0.51 (95% CI, 0.39–0.66; *P* < 0.001). For the purpose of controlling the potential confounding factors in adjuvant RT effectiveness, the multivariable Cox proportional hazards regression analysis was further applied. After the prognostic analysis was adjusted for the following clinicopathological parameters: tumor size, tumor grade, PR status, age at diagnosis, race and married status, we observed postoperative RT was independently associated with better BCSS benefit (adjusted HR, 0.64; 95%CI, 0.49–0.83; *P* = 0.001). Moreover, the results also indicated that tumor size, age ≥ 70 years, negative PR expression and DSW (divorced, separated, widowed) marital status were risk predictors which independently associated with BCSS(Table 3).

**Table 3:** Univariate and multivariate prognostic analyses of BCSS in all patients

Characteristics	Univariate			Multivariate		
	HR	95%CI	*P*	aHR	95%CI	*P*
*Treatment groups*						
Non-RT	1.00			1.00		
RT	0.51	0.39–0.66	< 0.001	0.64	0.49–0.83	0.001
*Age of diagnosis, years*						
< 50	1.00			1.00		
50–59	1.08	0.58–2.03	0.803	1.11	0.59–2.10	0.746
60–69	1.46	0.83–2.57	0.192	1.51	0.84–2.68	0.166
≥ 70	3.51	2.12–5.80	< 0.001	3.05	1.80–5.17	< 0.001
*Era of diagnosis*						
1998–2004	1.00					
2005–2009	1.04	0.77–1.41	0.789			
2010–2015	0.88	0.55–1.41	0.606			
*Race*						
White	1.00			1.00		
Black	1.49	1.00–2.22	0.047	1.44	0.96–2.15	0.076
Other	0.52	0.30–0.91	0.022	0.70	0.39–1.20	0.213
*Marital status*						
Married	1.00			1.00		
Non-married	0.92	0.56–1.51	0.736	0.98	0.59–1.61	0.931
DSW	2.25	1.71–2.96	< .001	1.56	1.17–2.08	0.002
Unknown	1.90	1.02–3.55	0.045	1.36	0.72–2.56	0.339
*Laterality*						
Left	1.00					
Right	0.95	0.74–1.23	0.690			
*Tumor size (T stage), cm*						
≤ 1.0 (T1)	1.00			1.00		
1.1–2.0 (T1)	1.85	1.35–2.53	< 0.001	1.92	1.40–2.63	< .001
2.1–3.0 (T2)	2.95	2.05–4.24	< 0.001	3.02	2.09–4.36	< .001
*Tumor grade*						
I	1.00			1.00		
II	1.35	1.00–1.82	0.049	1.31	0.97–1.77	0.079
III/IV	1.95	1.05–3.63	0.034	1.91	1.02–3.59	0.043
unknown	1.19	0.85–1.68	0.305	1.16	0.83–1.64	0.390
*ER status*						
Positive	1.00					
Negative	1.65	0.78–3.53	0.120			
Unknown	1.24	0.83–1.85	0.300			
*PR status*						
Positive	1.00			1.00		
Negative	1.56	1.08–2.26	0.020	1.47	1.01–2.13	0.045
Unknown	1.37	0.95–1.98	0.100	1.19	0.82–1.72	0.368
*Chemotherapy*						
No/Unknown	1.00					
Yes	0.75	0.46–1.21	0.460			

*HR* hazard ration, *aHR* adjust hazard raion, *CI* confidence interval

In the propensity-matched cohort, the survival analysis of BCSS also showed a significant difference between the two groups (log-rank test, *P* = 0.039; Fig. 3B). The BCSS rate for RT group was marginally better than non-RT group. The 5-year BCSS was 98.85% (95%CI, 98.24–99.25%)in RT group and 94.93% (95%CI, 93.46–96.08%) in non-RT group. The 10-year BCSS was 95.96% (95%CI, 94.66–96.95%) in RT group and 94.93% (95%CI, 0.93.46–96.08%) in non-RT group.The 15-year BCSS rate was 93.82% (95%CI, 91.75–95.38%) in RT group and 92.02% (95%CI, 89.39–94.03%) in non-RT group. The univariate analyses also confirmed that the RT group indicated a significantly favorable prognosis (HR, 0.71; 95%CI, 0.51–0.98; *P* = 0.041; Table 4). After adjusted age, race, marital status and tumor size, the result of multivariable Cox analysis did not change substantially (adjusted HR, 0.66; 95%CI, 0.47–0.92; *P* = 0.014; Table 4).

**Table 4:** Univariate and multivariate prognostic analyses of BCSS after PSM

Characteristics	Univariate			Multivariate		
	HR	95%CI	*P*	aHR	95%CI	*P*
*Treatment groups*						
Non-RT	1.00			1.00		
RT	0.71	0.51–0.98	0.039	0.66	0.47–0.92	0.014
*Age of diagnosis, years*						
< 50	1.00			1.00		
50–59	0.81	0.30–2.17	0.678	0.91	0.34–2.45	0.919
60–69	1.52	0.68–3.39	0.311	1.71	0.75–3.88	0.152
≥ 70	2.73	1.32–5.62	0.006	2.83	1.32–6.05	0.002
*Era of diagnosis*						
1998–2004	1.00					
2005–2009	1.02	0.70–1.50	0.902			
2010–2015	0.87	0.48–1.57	0.649			
*Race*						
White	1.00			1.00		
Black	1.60	0.99–2.61	0.055	1.67	1.02–2.74	0.042
Other	0.55	0.26–1.19	0.130	0.68	0.31–1.47	0.325
*Marital status*						
Married	1.00			1.00		
Non-married	1.18	0.62–2.22	0.614	1.18	0.62–2.25	0.620
DSW	2.14	1.48–3.08	< .001	1.60	1.10–2.35	0.015
Unknown	1.99	0.94–4.22	0.072	1.66	0.78- 3.52	0.189
*Laterality*						
Left	1.00					
Right	1.03	0.74–1.42	0.879			
*Tumor size (T stage), cm*						
≤ 1.0 (T1)	1.00			1.00		
1.1–2.0 (T1)	2.09	1.38–3.18	0.001	2.19	1.44–3.33	< .001
2.1–3.0 (T2)	3.46	2.15–5.59	< .001	3.58	2.21–5.79	< .001
*Tumor grade*						
I	1.00					
II	1.12	0.76–1.67	0.550			
III/IV	1.80	0.78–4.13	0.168			
unknown	0.87	0.57–1.38	0.594			
*ER status*						
Positive	1.00					
Negative	1.84	0.68–5.00	0.228			
Unknown	1.19	0.71–1.82	0.598			
*PR status*						
Positive	1.00					
Negative	1.22	0.72–2.07	0.452			
Unknown	1.19	0.77–1.85	0.421			
*Chemotherapy*						
No/Unknown	1.00					
Yes	0.56	0.25–1.27	0.169			

*HR* hazard ration, *aHR* adjust hazard raion, *CI* confidence interval

**Fig. 4:**
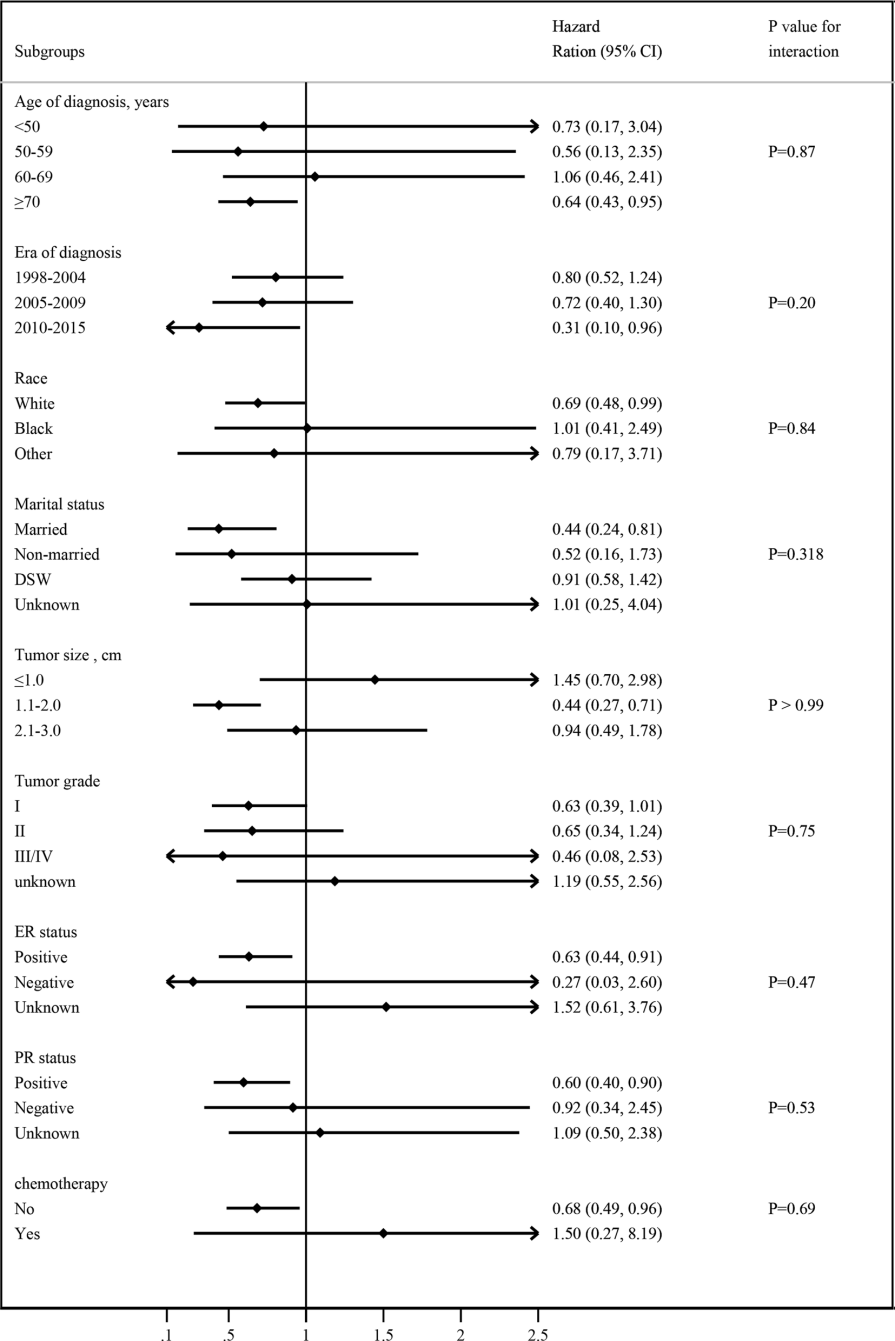
Forest plot depicting hazard ratios of adjuvant radiotherapy following lumpectomy versus lumpectomy alone for early-stage pure mucinous breast cancer in the propensity-matched population

The salutary effect of adjuvant RT on BCSS was further assessed in different subgroups among the matched population who underwent lumpectomy, and the HR interactions were tested (Fig. 4). The benefit of RT seemed to be significant in some patients. The HR was 0.64 (95%CI, 0.43–0.95) for patients aged 70 years and older, 0.44 (95%CI, 0.24–0.81) for married women, 0.44 (95%CI, 0.27–0.71) for patients with 1.1–2.0 cm tumor size, 0.63 (95%CI, 0.44–0.91) for patients with positive ER disease, 0.60 (95%CI, 0.40–0.90) for patients with positive PR tumor, 0.31 (95%CI, 0.10–0.96) for patients diagnosed during 2010–2015. However, as we can see from the Fig. 4, there were no statistically significance in global test for interaction (*P* > 0.05).

## Discussion

Among women with early-stage breast cancer receiving lumpectomy, the addition of RT is a standardized treatment based not only on its benefit in reducing ipsilateral breast cancer recurrence, but also on its ability to significantly improve BCSS [27, 28]. In this large population-based study, by using matched approach among patients who received lumpectomy with T1-2N0M0 (T ≤ 3 cm) pure mucinous breast cancer, our result clearly indicated that adjuvant irradiation following lumpectomy was significantly associated with BCSS benefit. The cumulative 15-year BCSS rate was 94.39% for women with pure mucinous breast cancer received adjuvant RT after lumpectomy, and 91.45% for patients treated with lumpectomy alone (HR = 0.51; 95% CI, 0.39–0.66; *P* < 0.001; Table 3). After adjustment for potential confounding factors, it was translated that the relative reduction of breast cancer-special death was 34%, and the absolute risk reduction at 15 years was 1.8%. In addition, heterogeneity tests of the interaction term were not significant among the matched population, suggesting that the protective prognostic value of adjuvant RT were consistent among different populations.

Our research has several potential strengths. To our best knowledge, this is a large cohort used to evaluate the effect on postoperative RT following lumpectomy among patients with early-stage pure mucinous breast cancer. Our study only aims to patient with tumor size less than 3 cm, which minimizes the impact of endocrine therapy on results. Propensity score matching was generated to reduce the confounding factors, leading to the baseline was comparable between treatment groups. In addition, the heterogeneity of RT effect was tested in subgroup interaction, which further verified the benefit of BCSS was attributable to radiotherapy rather than a baseline imbalance in clinicopathologic features.

Only a few studies have assessed the role of postoperative RT in this special type of breast cancer. Histological types of breast cancer, as prognostic risk factors, have rarely been evaluated in randomized trials related to radiation therapy [29]. Single-center experiences did not demonstrate that adjuvant RT improve recurrence free survival among patient with pure mucinous breast cancer [11]. In several Single-center retrospective studies, they were also failed to show that receiving adjuvant RT could improve the OS or DFS in pure mucinous breast cancer [1, 3, 6, 11, 19, 30, 31]. These negative results may be related to small sample sizes and limited follow-up periods in retrospective studies.A previous SEER analysis including 11,422 patients with pure mucinous breast cancer between 1973 and 2002, with a mean follow-up period of 84 months, showed that the addition of radiotherapy was not significantly asscosiated with prognosis using multivariable Cox regression analysis [3]. On the contrast, another SEER database study, including 8048 I-IV stage pure mucinous breast cancer from 2004 to 2014, found that radiotherapy was an independent factor for both OS and BCSS [22]. The opposing results may be related to the rapid development of radiotherapy and breast conserving surgery in the 1990s. A recent SEER research presented that radiotherapy following lumpectomy improved BCSS in pure mucious breast cancer patients aged ≥ 65 years diagnosed with T1–2N0 [23].Here, we assessed BCSS benefit of adjuvant RT following lumpectomy compared with lumpectomy alone in T1-2N0M0(tumor size ≤ 3 cm) pure mucinous breast cancer by using propensity score matching method and multivariable Cox regression analysis. Combined with the above, we believe that adjuvant RT is a value option for patients underwent lumpectomy with pure mucinous breast cancer, even in those with low-risk factors.

In the cohort, the risk prediction stratified score basing on clinical features and molecular biomarkers is low among patients with pure mucinous breast cancer, which might explain why absolute reductions in 15-year risk of breast cancer death tend to be modest. Besides, we believe patterns of intrinsic tumorigenesis of pure mucinous breast cancer may contribute to the result. This special type of breast cancer is distinct from other ER-positive/HER2-negative form of breast cance in terms of the tumorigenicity of mutated genes, suggesting that the genomic profiling of unusual variants of breast cancer should be taken into account in developing suitable personalized management for patients [5]. The PIK3CA mutation rate is 30–40% in ER-positive invasive ductal carcinoma and 7% in pure mucious breast cancer. The p53 mutation rate is 20% in ER positive invasive ductal carcinoma, but only within 5% in pure mucinous breast cancer. The probability of 1q gains and 16q losses is 10% in pure mucinous breast caner, which is 50% lower than that of ER-positive invasive ductal carcinoma [16]. In addition, pure mucious breast cancer had a relatively lower percentage of high 21-gene recurrence score patients than the infiltrating ductal carcinoma [32]]. In the future, for those with specific types of breast cancer, it is required to further study the prediction of clinical benefit from radiation therapy, and the identification of low-risk patients in whom radiation can be safely omitted.

Nevertheless, we must acknowledge several limitations of this study. There are inherent biases in retrospective study inevitably. The SEER database at present cannot provide the code on surgical margins, lymphovascular invasion, Ki-67 and hormone therapy. Data are missing in some cases for fundamental variables such as tumor size, grade, TNM stage, hormone receptor status. Fortunately, missing data in TNM stage and tumor size less than 5% of the total data. The radiotherapy treatment was not assigned at random. Although the propensity score matching method is efficient for reducing the confounding bias, a significant proportion of samples are censored in the paired matching process.

## Conclusion

In patients with localized pure mucinous breast cancer receiving lumpectomy, our results indicated that the management with adjuvant RT slightly improved BCSS compared with its omission. The adjvuant radiotherapy is an appropriate therapeutic option for patients received lumpectomy with localized pure mucinous breast cancer.

## Abbreviations

RT: Radiotherapy
SEER: The surveillance, epidemiology, and end results database
PSM: Propensity score matching
BCSS: Breast cancer-specific survival
PR: Progesterone receptor
ER: Estrogen receptor
HER-2: Human epidermal growth factors 2
DFS: Disease-free survival
OS: Overall survival
AJCC: American joint committee on cancer
HR: Hazard ratio
95%CI: 95% Confidence interval
